# Mind the gap: challenges in the clinical management of Lynch syndrome families

**DOI:** 10.1186/1897-4287-9-S1-P16

**Published:** 2011-03-10

**Authors:** Carin R Huizenga, Arelis E Martir-Negron, Kathleen R Blazer, Julie O Culver, Deborah J MacDonald, Jeffrey N Weitzel

**Affiliations:** 1 Clinical Cancer Genetics, City of Hope National Medical Center, Duarte, CA, 91010, USA

## Background

Lynch syndrome is associated with mismatch repair (MMR) gene mutations and high risk for early onset colorectal (mean age 44 yr), endometrial (46), ovarian (43), and stomach (56) cancers. The NCCN guidelines recommend colonoscopy in unaffected individuals with Lynch syndrome beginning at age 20-25 or ten years earlier than the youngest diagnosis in the family. Genetic testing is usually recommended just prior to the initiation of screening and not before age 18. Despite the reported mean ages, we often observe an outlier—an individual diagnosed with cancer much earlier than others in the family—which challenges our conventional wisdom and advice for the whole family. The purpose of this study was to explore how often this occurs, the size of age gap between the youngest diagnosis and the second youngest, and how these diagnoses may impact recommendations for other family members.

## Methods

Full pedigrees of participants with MMR mutations from an IRB-approved hereditary cancer registry were analyzed with special attention to age of cancer diagnosis and degree of relationship to the consultant. Cancer diagnoses included in analysis were those for which there are management recommendations (colorectal, endometrial, ovarian, gastric, small bowel).

## Results

Fifty-two Lynch syndrome families were identified; in 24 (46.2%), the earliest diagnosis was <35 and in 3 (5.8%) the earliest diagnosis was <28. Forty-eight had more than one individual with a Lynch-related tumor, and of those, 15 (31.3%) had an age gap of ≥10 years between the youngest diagnosis and second youngest diagnosis which would potentially impact recommendations for earliest age to test and screen. The average age gap overall was 7.3 years.

## Conclusions

These data represent a clinical conundrum for those who provide genetic cancer risk assessment. Many clinicians may be inclined to provide reassurance to Lynch families whose cancer diagnoses are later in age. However, our results suggest adherence to the guideline recommending relatively early age for initiation of testing and screening given that 31.3% of families in our sample had an individual diagnosed ≥10 years before the next youngest diagnosis. Conversely, “the ten year rule” would cause 46% of families in our sample to begin screening before age 25 even if most others in the family were diagnosed after 40, and would invoke screening as well as genetic testing prior to age 18 in 5.8% of families. More research must be done to elucidate causes of variable expressivity in this syndrome.

